# Mother–Offspring Bonding after Calving in Water Buffalo and Other Ruminants: Sensory Pathways and Neuroendocrine Aspects

**DOI:** 10.3390/ani14182696

**Published:** 2024-09-17

**Authors:** Daniel Mota-Rojas, Cécile Bienboire-Frosini, Agustín Orihuela, Adriana Domínguez-Oliva, Dina Villanueva García, Patricia Mora-Medina, Alex Cuibus, Fabio Napolitano, Temple Grandin

**Affiliations:** 1 Neurophysiology, Behavior and Animal Welfare Assessment, Department of Animal Production and Agriculture, Universidad Autónoma Metropolitana, Xochimilco Campus, Mexico City 04960, Mexico; 2 Department of Molecular Biology and Chemical Communication, Research Institute in Semiochemistry and Applied Ethology (IRSEA), 84400 Apt, France; 3 Facultad de Ciencias Agropecuarias, Universidad Autónoma del Estado de Morelos, Cuernavaca 62209, Mexico; 4 Division of Neonatology, Hospital Infantil de México Federico Gómez, Mexico City 06720, Mexico; 5 Facultad de Estudios Superiores Cuautitlán, Universidad Nacional Autónoma de México (UNAM), Cuautitlán Izcalli 54714, Mexico; 6 Faculty of Animal Science and Biotechnologies. University of Agricultural Sciences and Veterinary Medicine of Cluj-Napoca, 400372 Cluj-Napoca, Romania; 7 Scuola di Scienze Agrarie, Forestali, Alimentari ed Ambientali, Università degli Studi della Basilicata, 85100 Potenza, Italy; 8 Department of Animal Science, Colorado State University, Fort Collins, CO 80526, USA

**Keywords:** imprinting, sensitive period, mother–young recognition

## Abstract

**Simple Summary:**

This paper aims to review the development of the cow–calf bonding process in water buffalo (*Bubalus bubalis*) via olfactory, tactile, auditory, and visual stimuli. It will also discuss the neuroendocrine factors motivating buffalo cows to care for the calf. To develop cow–calf bonding, several olfactory, tactile, auditory, and visual cues need to be interchanged and processed by both the mother and the newborn. Sniffing, licking, grooming, and listening to the calf’s vocalizations are the first inputs during calving. Oxytocin is the main hormone that mediates selective maternal behavior. Understanding the importance of the sensitive period and the endocrine changes required to elicit maternal behavior could help provide the appropriate stimulus to both the buffalo cow and the calf during calving.

**Abstract:**

The cow–calf bonding is a process that must be developed within the first six hours after calving. Both the buffalo dam and the newborn calf receive a series of sensory cues during calving, including olfactory, tactile, auditory, and visual stimuli. These inputs are processed in the brain to develop an exclusive bond where the dam provides selective care to the filial newborn. The limbic system, sensory cortices, and maternal-related hormones such as oxytocin mediate this process. Due to the complex integration of the maternal response towards the newborn, this paper aims to review the development of the cow–calf bonding process in water buffalo (*Bubalus bubalis*) via the olfactory, tactile, auditory, and visual stimuli. It will also discuss the neuroendocrine factors motivating buffalo cows to care for the calf using examples in other ruminant species where dam–newborn bonding has been extensively studied.

## 1. Introduction

In mammals, the female mainly provides newborn care via exclusive and preferential behavior towards the offspring [1,2,3,4]. Cow–calf bonding is an early learning process where the offspring and the mother establish a social preference or selective bond with each other [5,6,7]. It is developed immediately after calving, the so-called “sensitive period” [8]. In ruminants such as the water buffalo (*Bubalus bubalis*), the first six hours after calving cover this period where cow–calf bonding must be developed to ensure the survival and growth of the newborn [8,9,10,11]. Due to limited studies on water buffalo, research on other ruminant species will be reviewed. This is important to stimulate both discussion and further research on water buffalo. For the domestic water buffalo, an important milk-producing species with over 205 million head worldwide, promoting adequate maternal care immediately after calving is essential for the dairy industry [12]. It is estimated that 13% of the global milk yield corresponds to water buffalo, particularly in Asian countries (97%) [13,14]. Buffalo milk is characterized as a high-quality product compared to cow’s milk with higher values for fat (70 ± 6 vs. 41 ± 1 g/kg) [15,16]. For dairy animals, the first hours after calving are essential to develop cow–calf affiliative behaviors, not only from a productive perspective but also to improve both the calf and dam’s welfare [17]. 

It has been stated that providing selective maternal care for the offspring can reduce neonatal mortality rates by up to 19.5% in the first days [18,19,20,21] and up to 84% during the first month after calving [22]. Buffalo calves, as precocial newborns, are born with a fully developed motor system that stimulates them to stand up shortly after calving to prevent predation [23,24]. Therefore, the anatomical and functional development of ruminants at birth can influence maternal care and cow–calf bonding [11,25,26]. 

Bonding requires the integration of several sensory cues in some cerebral structures [7,27,28,29]. One of the first approaches between the dam and the newborn calf is via vocalizations and the newborn odor of the placental fluids and the perianal region [23,24,30,31]. Eating the placenta or placentophagy is an activity observed in ungulates at the end of parturition [11,32,33,34]. Placentophagy has several benefits for both the newborn and the dam. On one hand, eating the placenta increases the action of brain opioids involved in maternal care and endogenous analgesia while improving the dam–calf bonding [35,36]. Hence, the olfactory cues promote tactile stimulation via licking to remove the placental membranes, prevent heat loss, and stimulate the breathing function [37]. It has been reported that 100% of buffalo females lick and groom their offspring during the first six hours after calving [38,39]. Grooming also strengthens the cow–calf bonding by encouraging the newborn to search for the udder and suckle colostrum for passive immunity transfer [40,41,42]. Moreover, suckling establishes positive feedback between cow–calf interaction and the endocrine control of maternal behavior through oxytocinergic pathways [29,43,44,45].

To process these sensorial inputs, cerebral structures such as the locus coeruleus, olfactory bulb, auditory cortex, visual cortex, and limbic system interact with endocrine modulators of maternal behavior, particularly the oxytocinergic system [11,26,46,47]. To date, limited studies have focused on studying maternal bonding in water buffalo. Due to the complex integration of the maternal response towards the newborn, this paper aims to review the development of the cow–calf bonding process in water buffaloes via the olfactory, tactile, auditory, and visual stimuli. The present review will discuss the neuroendocrine factors motivating buffalo cows to care for the calf, using examples in other ruminant species where dam–newborn bonding has been extensively studied, to feed the discussions. 

## 2. Sensitive Period for the Establishment of Maternal Recognition in Ruminants

**Figure 1:**
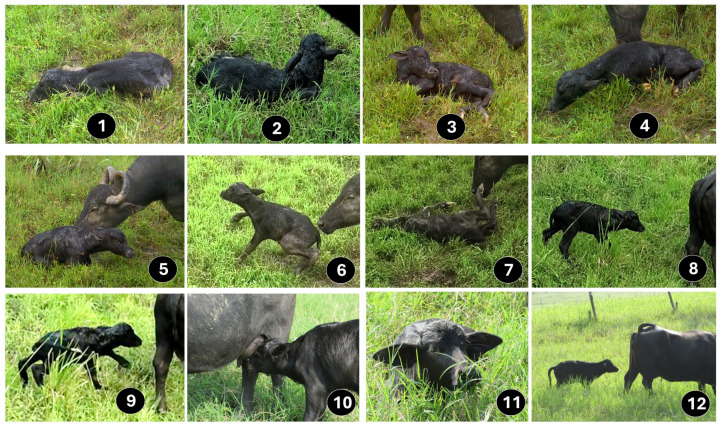
Sequence of events associated with the performance of the newborn buffalo 1. First inspiration. 2. First ear movement. 3. First head movement. 4. First attempt to stand. 5. Ventral decubitus position. 6. Standing for the first time. 7. First fall. 8. Neonate standing without falling. 9. Walking. 10. First contact with the udder. 11. First rest after nursing. 12. Following the dam.

The “sensitive period” is known as the period where preferential or exclusive care for the newborn is established via cow–calf bonding [47,48]. Since this period requires the integration of both the dam’s responses and the calf’s learning ability [30], it is considered a learning process where hormonal inputs and neuronal plasticity encourage maternal care [49]. Filial recognition in ruminants requires an appropriate calf’s vitality so the newborn can respond to the stimulus coming from the female (Figure 1) [11,23,50]. For example, buffalo calves make their first attempts to stand up within the first 27.9–214 min after calving and suckle in an average of 212.0 ± 110.0 min [38,51]. These behaviors are facilitated with tactile stimulation of the buffalo dam, which starts grooming the calf within the first 45 min and encourages standing up [38].

In gregarious species such as ruminants, the sensitive period starts immediately after parturition and can last up to six hours [9,10]. Once the bonding is developed, the dam actively rejects other calves and exclusively provides nutrition and protection to the newborn [20,52,53]. The establishment of maternal recognition starts before calving [44], where stimulation of the vaginocervical region stimulates sympathetic pathways to release oxytocin (OT) and initiate maternal behaviors [11,54].

Interferences during the sensitive period (e.g., human intervention) are critical for the calf’s development [55]. Some studies in bovines have reported that five minutes of interaction immediately after calving is enough to develop the maternal bond while separation for five hours after the calving causes maternal rejection in 50% of the cases [11,56]. 

**Figure 2:**
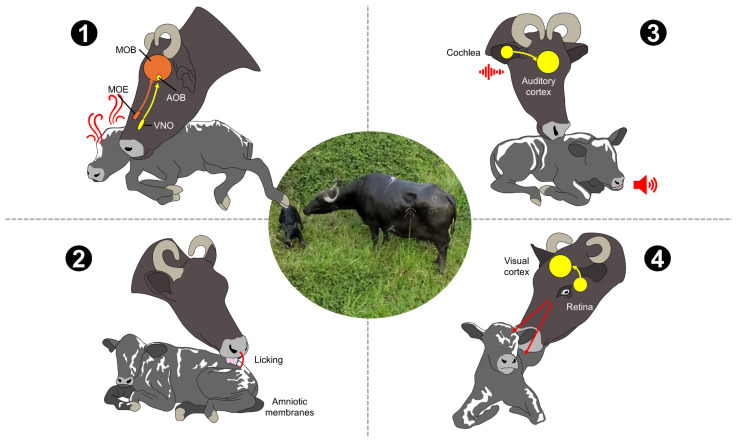
Sensorial stimulation between the dam and the newborn after calving. 1. Olfactory stimulation, provided by the fetal membranes and amniotic fluid is recognized by the vomeronasal organ (VNO), the main olfactory epithelium (MOE), the accessory olfactory bulb (AOB), and the main olfactory bulb (MOB). 2. Tactile stimulation includes behaviors such as licking and grooming. 3. Auditory stimulation recognizes vocalizations from both the mother and the calf. 4. Visual stimulation includes the visual recognition of the newborn calf. All these sensorial cues establish the selective cow–calf bond.

During the sensitive period, different sensorial stimulations are exchanged between the buffalo cow and the calf [8,38,57]. Mutual recognition requires processing olfactory, tactile, auditory, and visual stimuli into a neuroendocrine response (Figure 2) [8,11,26]. The activation of these communication pathways causes behavioral changes in both the mother and her offspring [23,58,59]. For example, after calving, buffalo dams lick the newborn to encourage them to stand up and consume colostrum [39,60,61]. In mammals, these changes are mainly mediated by OT and will be discussed in the next sections [8,62,63]. 

## 3. Sensorial Stimulation Required for the Cow–Calf Bonding 

### 3.1. Olfactory Stimulation

**Figure 3:**
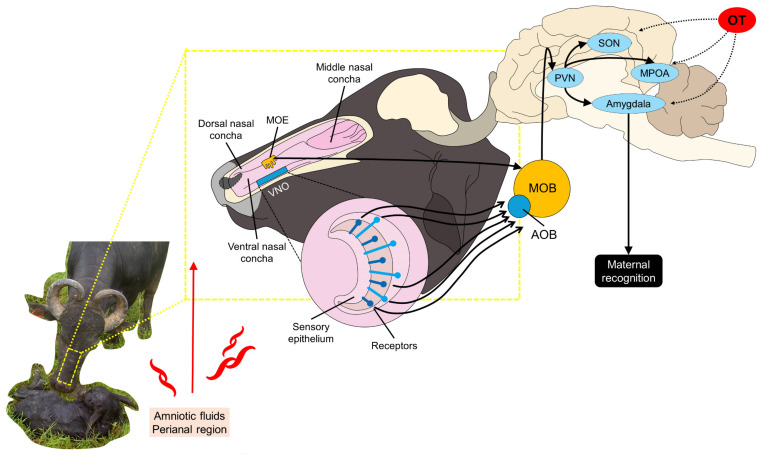
Neurophysiology olfactory stimulation during calving. Olfactory stimulation starts immediately after calving by detecting the amniotic fluids and odor of the newborn, particularly in the perianal region. The sensory epithelium and receptors in the vomeronasal organ (VNO) and the main olfactory epithelium (MOE) project the information to the accessory olfactory bulb (AOB) and the main olfactory bulb (MOB), respectively. To develop maternal recognition, structures such as the paraventricular nucleus (PVN), supraoptic nucleus (SON), medial preoptic area (MPOA), and the amygdala are required to process and integrate the olfactory cues.

Olfactory signals are considered one of the essential and most selective mechanisms that ruminants have to recognize their offspring [64,65,66]. Olfactory inputs are mainly integrated by the vomeronasal organ, the main olfactory epithelium, the accessor olfactory bulb, and the main olfactory bulb (Figure 3) [67,68,69]. In general, ruminants are highly receptive to the calf’s odor due to the neuronal plasticity in the vomeronasal organ [70], and dams use this sense to localize the newborn in a close range [39,71,72]. In ewes, a distance below 0.25 m is considered adequate to identify the lamb. Therefore, it is important to maintain maternal proximity in ruminants in the first days after parturition [73]. In the same species, it has been reported that after lambing, two to four hours are required to establish a selective olfactory discrimination of the offspring [9,74,75].

Immediately after calving, buffaloes spend much time sniffing the perineal area [51]. In other ruminants, such as ewes, a preference for the perineal area has also been observed, probably due to the presence of chemical substances recognized by the dam [50,76,77,78]. Dubey et al. [51] and Lanzoni et al. [38] have reported in Surti and Italian Mediterranean buffaloes that the dams sniff and lick their calves mainly during the first day.

It has been mentioned that multiparous dams sniff their offspring more frequently than primiparous animals, probably due to maternal inexperience in the latter [33,53]. Moreover, the calves from dams that groomed their offspring showed a low incidence of maternal rejection and had higher daily weight gains. That shows the maternal bonding effect on cow–calf interaction [38]. Sniffing and licking the amniotic membranes also promotes cow–calf bonding and encourages breathing and eliminatory behaviors (e.g., defecation and urination) [50,79,80,81]. 

Due to the importance of olfactory bonding at birth, some studies on ruminants have reported the effect of anosmia on the presentation of maternal behaviors. For example, when performing olfactory bulbectomy or resection of olfactory nerves to ewes, authors have found that anosmic ewes do not show selective maternal care and indistinguishably accept suckling from filial and non-filial lambs [82,83,84]. Other studies in the same species have shown that suppression of olfactory neurogenesis in ewes affects mother–young bonding and ewes tend to reject the newborn [67]. Moreover, the endocrine response (e.g., OT) also participates and needs to activate brain regions required for affiliative behaviors. Therefore, olfactory recognition of the newborn requires the neuroendocrine integration of the stimulus, which will be discussed in the next section. 

### 3.2. Tactile Stimulation

Tactile stimulation is started by the dam via physical contact with the newborn calf [11,85]. Licking the calf is one of the main maternal behaviors that ruminants show immediately after calving [86]. Licking the fetal membranes, or placentophagy, helps the newborn to stand up [11,32,33,34]. Precocial newborns, such as buffalo calves, are born with a fully functional locomotor system that can be stimulated by licking and interacting with the calf [38,87,88,89]. Attempts to stand up can start within the first 10 min after calving, and buffalo calves can successfully incorporate within 50 min [26,32]. 

**Figure 4:**
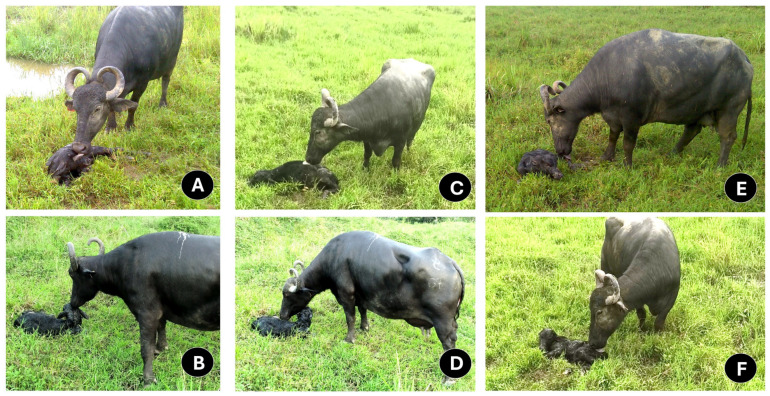
Body regions of the neonate where licking begins as an indicator of maternal behavior experience. (**A**,**B**), licking in the cranial region. (**C**,**D**), licking in the middle region. (**E**,**F**), licking in the caudal region.

Constant licking encourages breathing by cleaning the nasal passage [32,90]. As Figure 4 shows, grooming starts from the head, nose, and mouth—to prevent airway obstruction—and moves to the back, rump, and anogenital region [32,38,90]. Apart from motivating standing up, grooming also incites defecation and urination in newborn ruminants [39,51,91,92]. 

**Figure 5:**
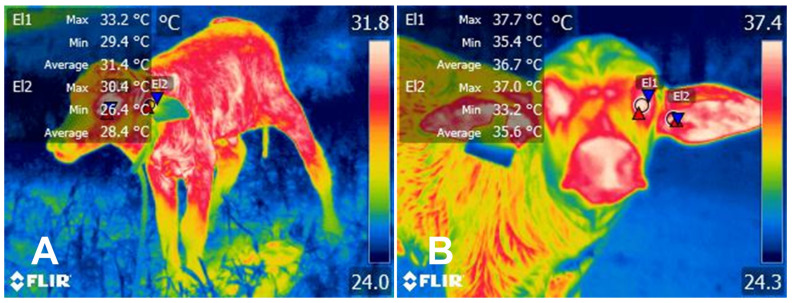
Preliminary results of thermal images of tactile stimulation effect on the newborn Murrah calf. (**A**) Non-licking calf. The average periocular (El1) and auricular (El2) temperatures of the calf are 31.4 and 28.4 °C, respectively. (**B**) Contrarily to the non-licking calf, the average periocular (El1) and auricular (El2) temperatures of the calf that received maternal attention are higher by 5.3 °C and 7.2 °C, respectively. This is associated with the newborn calves’ susceptibility to lose heat by evaporation of the amniotic fluid during calving. The images were taken with a FLIR thermal camera (E80, FLIR Systems, Orlando, FL, USA), positioned 1 m from the calf at an angle of 90°. Emissivity of 0.95, and IR resolution of 320 × 240 pixels.

Licking also prevents heat loss by removing placental membranes and drying the calf’s coat [34]. Preventing hypothermia is essential in newborns since low corporal temperatures might interfere with the newborn’s vitality and deplete limited glycogen reserves due to the activation of thermoregulatory pathways [93,94]. Figure 5 shows thermal pictures taken by the present authors, where preliminary results on the effect of licking were evaluated in buffalo calves. It can be observed that the periocular—a region correlated with core temperature—and the auricular average temperature are lower in the non-groomed calf by up to 7.2 °C. 

During the post-calving period, licking is performed by 100% of buffalo cows [33,39]. In Murrah, Surti, and Italian Mediterranean buffaloes, Lanzoni et al. [38] and Dubey et al. [51] reported active licking during the first six hours after calving. Grooming the newborn started within the first 11.8 ± 15.0 min [38]. A study showed that buffalo dams lick and sniff the newborn for approximately 11.54–13.15 min during the first half hour after calving, spending an average of 4.01 ± 0.85 min in the anogenital region [51]. Later, the time licking the newborn calf decreases up to 0.13 ± 0.06 min at 24 h [51], similar to what was reported in Italian Mediterranean buffaloes, in whom most of the tactile interactions between the cow and the calf (grooming) occur in intervals of 7.7 ± 2.5 min to later decrease to 1.9 ± 1.4 min at 24 h post-calving [38]. Grooming is particularly reported in multiparous dams due to their maternal experience [33]. 

Tactile stimulation also encourages the calf to search for the udder for colostrum intake [34,38,51]. Some authors mention that thermal inputs are also involved in the localization of the udder to start suckling [23]. Likewise, licking has been shown to decrease anxiety, as observed in 30 buffalo cows [39]. Moreover, since the dam’s saliva contains chemosignals, the newborn recognizes the mother’s odor and vice versa, integrating olfactory and tactile cues [90]. This is associated with the hormonal control of maternal behavior, mainly by OT and vasopressin [95,96]. Grooming the newborn initiates an oxytocinergic cascade that reinforces maternal behavior and, thus, cow–calf bonding, as observed in dairy cattle [97]. The endocrine modulation will be explained further in the manuscript; however, the suppression of OT release using epidural blocks has been shown to inhibit or significantly reduce licking behavior in heifers [98]. 

### 3.3. Auditory Stimulation

Auditory cues include vocal communication between the buffalo cow and the calf [23]. Newborn ruminants can vocalize within the first hours after parturition to call for the dam [33,51]. Research in calves and lambs has shown that even 2-day-old newborns can identify vocalizations from their mothers and respond to vocalizations to promote early interactions [99,100,101]. In sheep, vocal cues coming from the dam are frequently heard during the first three hours after lambing [77]; after this time, ewe–lamb auditory stimulation gradually decreases over the first 24 h after lambing [102]. 

Research regarding the importance of auditory stimulation between buffalo cow–calf is limited. However, studies on other ruminants have shown some preferences for calves over certain sounds. For example, in the study by Barfield et al. [100], Holstein’s calves were exposed to recordings of their mother’s call vs. a non-mother speaker. The authors found that calves spent significantly more time in front of the speaker that contained their mother’s call (an average of 45–50 s) and maintained the closest proximity when hearing it (an average of 1 m). This study highlights the ability of the calves to learn and distinguish their mother’s vocalization in 3–5-week-old calves [100]. Moreover, it also emphasizes that calves do not vocalize when separated from the dam, showing the importance of the cow–calf interaction through auditory cues. 

A similar alteration in the degree of vocal response has been observed in Holstein–Friesian cows when separated from their calves by Green et al. [103]. When the cow–calf interaction was maintained, cows emitted a higher number of closed-mouth calls (149.6 ± 23.1 calls) while simultaneously licking the calf. Contrarily, during calf separation, cows emitted more frequently open- and mixed-mouth calls (8.03 ± 4.34 and 23.19 ± 7.84 calls, respectively) with a shorter interval time (5.93 ± 0.51 s vs. 7.59 ± 0.66) and became more alert as a stress-associated response. Behavioral observations were also recorded in free-range *Bos taurus* cattle when exposed to playbacks of their calf’s calls. When listening to the recordings, cows were more likely to respond to their own calf’s calls with ear movements or look towards the speaker, as well as walking towards the sound source [104]. This suggests that cow–calf bonding requires bidirectional auditory connections. 

### 3.4. Visual Stimulation 

There is limited information regarding the importance of visual stimulation in water buffaloes. However, it is known that vision is the main sensory organ for ruminants to establish and maintain the cow–calf bonding [23,105]. Studies performed in Taurine crossbred dams have evaluated that cow–calf separation with and without a visual barrier alters the behavior and other maternal communication pathways (e.g., auditory stimulus) [106,107]. Johnsen et al. [107] mentioned that maintaining physical and visual contact between the dam and the calf during maternal separation reduces the responses associated with distress, such as fewer high-pitched vocalizations, and maintains normal milk intakes. 

**Figure 6:**
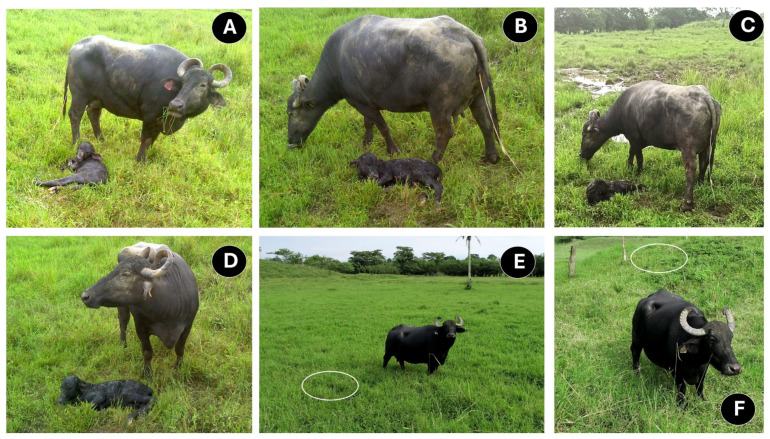
Pictures of water buffalo behaviors that might be regarded as a lack of an appropriate cow–calf bonding. (**A**–**C**): Buffaloes that initiated trophic behavior immediately after calf expulsion. (**D**–**F**): Buffaloes indifferent to the birth of their calf. The cow–calf bond was not established, and they initially rejected their calf. The presentation of these reactions could be associated with dystocia, pain, and maternal inexperience.

When maternal bonding is not appropriately developed between the buffalo cow and the newborn calf, negative behaviors such as aggression, rejection, or feeding restriction can be observed (Figure 6) [23]. Yadav et al. [33] observed that only 1 of 20 buffalo cows rejected her calf. However, in cattle, authors such as Barrier et al. [108] and von Keyserlingk and Weary [53] emphasized that the risk of calf rejection increases after dystocia due to the pain and the physiological challenge that it represents. Therefore, it is essential to consider that, during the sensitive period, an appropriate sensory interchange must be encouraged immediately after calving.

## 4. Neuroendocrine Control of Maternal Bonding

The cow–calf bonding process requires the interaction between the sensory cues coming from the animals, the neuroendocrine responses, and the integration of this information in several brain structures that are essential for maternal behavior [23]. 

### 4.1. Main Neurotransmitters and Hormones Related to Maternal Behavior

Several hormones and neurotransmitters are necessary to trigger and maintain maternal behavior and mother–young recognition in ruminants [10]. Among these, OT, progesterone, estradiol, and prolactin (PRL) are the main modulators of affiliative behaviors [43,44,53,109]. Likewise, neurotransmitters such as glutamate and dopamine also contribute to maternal bonding [110]. 

**Figure 7:**
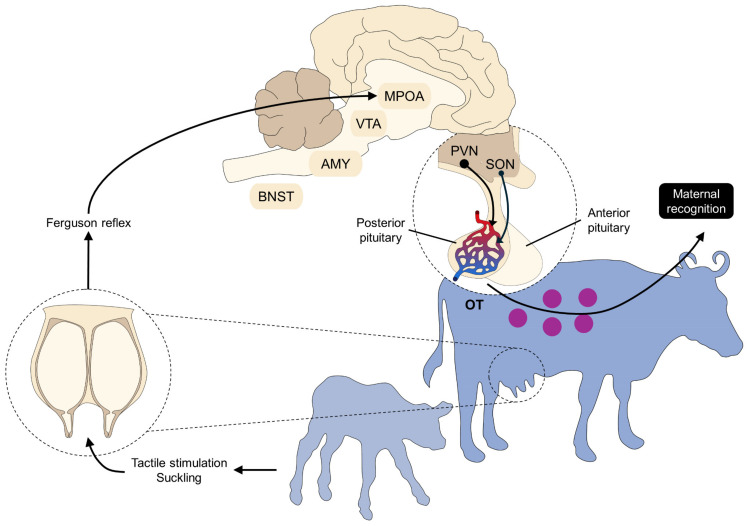
Endocrine control of cow–calf bonding: oxytocin. Maternal recognition requires the integration of sensory cues with the corresponding neuroendocrine response. Sensory stimuli are processed in the brain, mainly by the medial preoptic nucleus (MPOA), ventral tegmental area (VTA), amygdala (AMY), and bed nucleus of the stria terminalis (BNST). Neurons project to the paraventricular (PVN) and supraoptic nucleus (SON) of the pituitary, initiating the release of maternal-related mediators such as oxytocin (OT). Oxytocinergic pathways are also mediated by positive feedback triggered by the newborn calf. Via the Ferguson reflex, tactile stimulation by the calf—suckling—promotes OT release, enhancing maternal and cow–calf bonding.

In particular, the oxytocinergic system is the most relevant endocrine modulator of the mother–young interaction in mammals [111]. Another aspect where OT participates is lactation and milk ejection [10]. The supraoptic and the paraventricular nuclei of the hypothalamus produce OT to be stored and released by the posterior pituitary [112]. The activation of the oxytocinergic neurons is initiated during parturition due to the so-called Ferguson reflex [44,59,113,114]. Stimulation of the mechanoreceptors in the cervicovaginal region projects sensory information to the hypothalamus to release OT to initiate uterine contractions and participate in maternal behavior (Figure 7) [115].

Studies evaluating the concentration of OT before and after calving have found significant increases, as reported by López-Arjona et al. [116] in Holstein cows. In these animals, salivary OT concentrations were higher immediately after calving (1105.0 pg/mL) and progressively decreased to 7.1 pg/mL after one week [116]. Plasma OT levels of the jugular and utero-ovarian veins of cows also increased to 77.4 ± 19.1 μU/mL and 91.6 ± 21 μU/mL during calving, in contrast to an average concentration of 0.77 ± 0.1 μU/mL one week before calving [117]. Similarly, the number of OT receptors in the endometrium and cotyledons of cattle increased during parturition (7300 ± 1418 fmol/mg and 163 ± 36 fmol/mg, respectively) [118]. These changes are associated with the sensitive period within the first six hours, where OT and oxytocinergic receptors are required to establish maternal preference [77,119]. 

On the other hand, although PRL is mostly associated with milk production, it also participates and interacts with OT to promote maternal behavior, activating regions in the forebrain [120]. This cerebral region processes olfactory cues and integrates maternal responses dependent on PRL. In goats, it was reported that PRL concentrations increase when interacting with their kid, in contrast to the deleterious effect of an alien goat [121]. Therefore, PRL also modulates maternal selectivity, and a lack of appropriate levels of PRL might impair maternal recognition in the sensitive period [122].

### 4.2. Neurostructural Pathways to Establish Cow–Calf Bonding

Mutual recognition between the mother and the newborn requires the activation of brain structures to respond to multimodal sensory signals and integrate the maternal response [123,124]. These cerebral regions include the locus coeruleus, amygdala, hippocampus, cerebral cortex, medial preoptic area, and the olfactory, auditory, and visual cortices [58,125,126,127]. Additionally, the nucleus accumbens, ventral tegmental area, lateral septum, and bed nucleus of the stria terminalis participate during the cow–calf bonding [63]. In particular, the amygdala, mesencephalon, ventral tegmental area, nucleus accumbens, olfactory bulb, hippocampus, and anterior cingulate are critical for the sensitive period due to the significant amount of OT receptors and their sensitivity to OT [44,125,126]. Moreover, the sensory cues participate in other maternal-related care, such as milk ejection by medial preoptic area activation, the paraventricular nucleus, and the supraoptic nucleus [128,129,130,131,132].

Since it is a process that requires neuroplasticity and learning, the activation and interconnection between these regions differ according to the stimuli type (e.g., olfactory, tactile, auditory, or visual) [11,23,46,124]. For example, to develop olfactory memory in the ewe, the odor signals from the calf need to be processed by the vomeronasal organ or the main olfactory epithelium and its projections to the piriform cortex, orbitofrontal cortex, hippocampus, hypothalamus, and amygdala [126]. In the olfactory bulb of mammals, neurotransmitters such as glutamate and gamma amino butyric acid (GABA) respond to the activation of the sensory fibers in the vomeronasal organ and the main olfactory epithelium to the amniotic fluids [133,134,135]. The neural control of the olfactory stimuli after calving might involve the remodeling of neuronal pathways to process olfactory signals. The early learning ability of the dam and the newborn establishes neuronal interconnections to accept and recognize each other, the so-called olfactory memory [67]. In lambs, studies have shown that the maturity of these neurons is exacerbated during the sensitive period, where connections in the olfactory bulb respond to the presence of the lamb [66,77,134]. 

The hippocampus also participates in processing tactile stimulation to produce emotional responses [136,137,138]. Affiliative behaviors such as grooming and emotional valence are mediated by the nucleus accumbens, the limbic system, and the dopaminergic reward system of mammals [23,139]. In the case of auditory stimulation, the ungulate’s auditory cortex and the paraventricular nucleus are essential to perceive, integrate, and respond to vocal cues coming from both the dam and the newborn [26,105,140,141]. Finally, visual cues are processed by the optic nerve and its connections with the occipital lobe, the lateral geniculate nucleus, and the temporal cortex. 

## 5. Human Intervention and Weaning: Aspects That Can Influence Maternal Bonding 

During and after calving, certain events can damage cow–calf bonding, including human intervention [23,142]. This effect has been mostly studied in *Bos taurus* cattle. Thus, although some inferences can be made from these reports, it is essential to investigate this in water buffaloes. 

Dystocia, or difficult calving, is one of the events that negatively affect cow–calf bonding, in particular when humans intervene. Edwards and Broom [143] reported that primiparous cows are reluctant to stand up after dystocia, interrupting physical contact with the newborn and possibly impeding maternal bonding. Similarly, Hogan et al. [144] mentioned that dystocia cases in cattle might increase neonatal calf mortality due to impaired nursing, compromising milk intake. 

Maternal instincts and communication with the newborn can also be affected by parity [145]. For example, failure to lick the newborn was observed in primiparous cows (3 of 38 cows), while no multiparous dams failed to lick the calf [146]. Moreover, agonistic behavior (aggression towards the calf) and less time licking the calf have been reported in primiparous cows separated immediately after calving [56]. In contrast, in Surti buffaloes, it has been reported that maternal behavior is correlated with neonatal responses but that parity does not significantly influence the dyad [51]. 

Interrupting cow–calf bonding at weaning is a routine practice in dairy farms that undoubtedly causes stress to the animals due to the social and nutritional dependence of the newborn on the dam [147,148]. Some studies have proposed prolonging the weaning periods (more than the established 8–12 weeks) or increasing the contact between the dam and the newborn to prevent the deleterious effects triggered by stress on the productive performance and health of animals. An example is Wenker et al. [147], who found that debonding Holstein calves that had entire contact with the dam for 49 days after calving resulted in lower daily weight gains (0.64 ± 0.08 kg/d) than calves with partial contact (0.81 ± 0.08 kg/d). Jensen et al. [148] highlight that strategies such as cow–calf contact are an alternative to conventional early weaning by gradually reducing the daily contact between the dam and the newborn to interrupt the mother–calf bond progressively. Recently, Vogt et al. [149] showed that this gradual weaning strategy could reduce weaning and separation distress in German Holstein calves.

Therefore, environments that promote natural behaviors and reduce stress will potentially promote adequate cow–calf bonding since even ambient factors such as season have been shown to influence calf mortality, as described by Moore et al. [150] in calves, in whom the highest risk of mortality was recorded during winter, the months with the highest rainfall. Although additional studies are required to establish the neurobiology of the importance of the environment, during the wettest months of the year, the wet coat of the calves might interfere with maternal bonding and make them prone to hypothermia, limiting their mobility and, thus, the interaction with the dam.

## 6. Conclusions

Immediately after calving, the next six hours are the sensitive period in which buffalo dams and the newborn calves need to establish a maternal bond so the dam can provide exclusive care for the calf. Olfactory, tactile, auditory, and visual cues from the buffalo cow and the calf must trigger a neuroendocrine response to develop this bond. In particular, OT is the main hormone associated with affiliative behaviors. Oxytocinergic pathways highly intervene in the processing of multisensorial signaling by projections to crucial cerebral structures such as the amygdala, mesencephalon, ventral tegmental area, nucleus accumbens, olfactory bulb, hippocampus, and anterior cingulate. Grooming the calf, sniffing and licking fetal membranes, recognizing vocalizations from filial individuals, and physical contact during the first hours after calving encourage maternal care and benefit newborn behavior and vitality. Understanding the importance of the sensitive period, as well as the endocrine changes required to elicit maternal behavior, could help to provide the appropriate stimulus to both the buffalo cow and the calf during calving.

## Data Availability

Data sharing is not applicable.
